# Optimal balance of efficacy and tolerability of oral triptans and telcagepant: a review and a clinical comment

**DOI:** 10.1007/s10194-011-0309-5

**Published:** 2011-02-25

**Authors:** Peer Tfelt-Hansen

**Affiliations:** Department of Neurology, Danish Headache Center, University of Copenhagen, Glostrup Hospital, Glostrup, Denmark

**Keywords:** Migraine, Acute treatment, Triptans, Dose–response curve, Telcagepant

## Abstract

Dose–response curves for headaches relief and adverse events (AEs) are presented for five triptans: sumatriptan, zolmitriptan, naratriptan, almotriptan, and frovatriptan, and the CGRP antagonist telcagepant. The upper part of the efficacy curve of the triptans is generally flat, the so-called ceiling effect; and none of the oral triptans, even in high doses, are as effective as subcutaneous sumatriptan, In contrast, AEs increases with increasing dose without a ceiling effect. The optimal dose for the triptans is mainly determined by tolerability. Telcagepant has an excellent tolerability and can be used in migraine patients with cardiovascular co-morbidity. Based on the literature the triptans and telcagepant are rated in a table for efficacy and tolerability.

We conclude that a single 6 mg dose of sumatriptan given subcutaneously is a highly effective, rapid-acting, and well-tolerated treatment for migraine attacks. [1]

## Introduction

The vignette suggests that “the philosophers’s stone” has been found with the introduction of sumatriptan. Subcutaneous sumatriptan 6 mg and subcutaneous naratriptan 10 mg are both highly effective drugs. Headache relief at 2 h was 81, 85–89% [1–4], and 91% [2], respectively; but in both cases there is a high incidence of adverse events (AEs) (53–71, 85% [2, 3]). Most of these AEs after subcutaneous sumatriptan were reported as being minor and transient in one study [1] whereas in another simultaneously conducted study 20% of the AEs after sumatriptan and 17% after placebo were described as severe [2].

In clinical practice with oral triptans not all migraine patients respond to a triptan and AEs can be a problem. The optimal balance of efficacy and tolerability depends on the combined dose–response curves for both antimigraine effect and incidence of AEs. These dose–response curves for oral triptans will be reviewed, the findings discussed and finally my clinical comments will be presented.

## Methods and results

Dose-defining, randomised, controlled trials (RCTs) of triptans were searched for in PubMed and in The Headaches [5]. Studies defining the dose–response curves of oral triptans for both efficacy and the incidence of AE were selected for analysis. In addition, large dose-defining studies on the CGRP antagonist telcegepant were searched for.

**Fig. 1:**
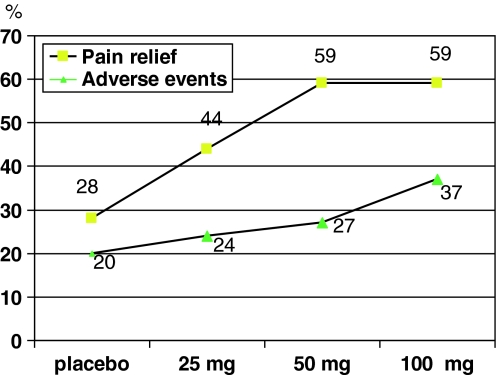
Effect of sumatriptan 25, 50, and 100 mg on headache relief and adverse events in one RCT [6]

**Fig. 2:**
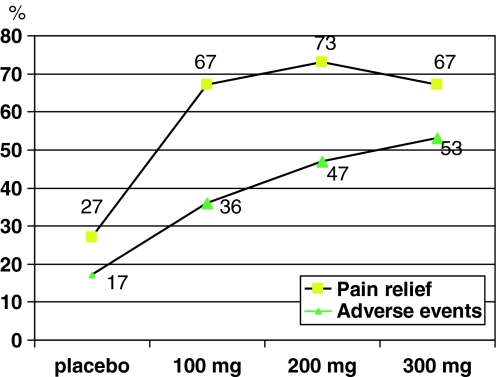
Effect of sumatriptan 100, 200, and 300 mg on headache relief and adverse events in one RCT [7]

**Fig. 3:**
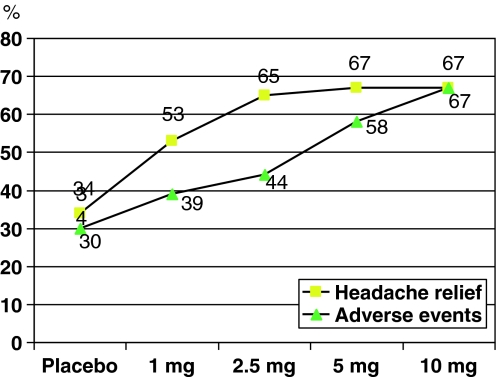
Effect of zolmitriptan 1, 2.5, 5, and 10 mg on headache relief and adverse events in one RCT [14]

**Fig. 4:**
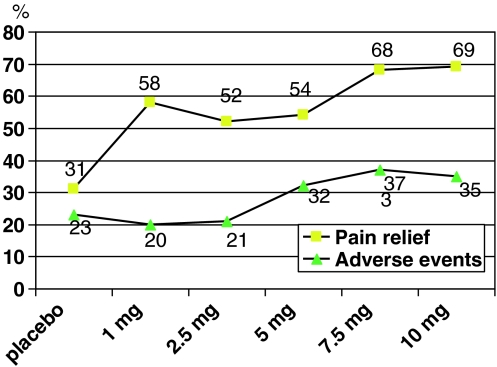
Effect of naratriptan 1, 2.5, 5, 7.5, and 10 mg on headache relief and adverse events in one RCT [16]

For three triptans (zolmitriptan, naratriptan, and almotriptan) the balance of efficacy and tolerability could be evaluated by drawing the curves from one dose-defining study as shown in Figs. 2, 3, and 4. Two dose-defining studies [5, 6] were needed to evaluate the full dose–responses curves for sumatriptan and frovatriptan (Figs. 1, 2, and 6). For rizatriptan and eletriptan the incidence of AEs was not presented [7–11] and only the results for efficacy of these two triptans are mentioned briefly.

Sumatriptan is the first and standard triptan and it took two studies, from 1991 and 1998, before the dose–response curve for oral sumatriptan could be established (Figs. 1, 2) [6, 12]. It is evident from Figs. 1 and 2 that there is an upper flat part of the dose–response curve for efficacy, starting at sumatriptan 50 mg, and there is no increase in efficacy up to the 300 mg dose. The incidence of AEs increases with increasing dose of sumatriptan, reaching a maximum of 53% after 300 mg sumatriptan. 25 mg sumatriptan was the minimum effective dose [6]. For sumatriptan 50 mg there was 7% more AEs than after placebo (Fig. 1a) which is quite similar to the 9% found in one meta-analysis [13]. The recommended starting dose of oral sumatriptan is 50 mg. This choice is based on maximal efficacy and reasonable tolerability (Figs. 1, 2).

The dose–response curves for zolmitriptan are shown in Fig. 3 [14]. Again there is a flat upper part for efficacy. The starting dose for this plateau is 2.5 mg zolmitriptan. The AEs increase with increasing dose and reach a maximum of 67% after 10 mg zolmitriptan. For zolmitriptan 2.5 mg there were 14% more AEs than after placebo. This incidence is quite similar to the 15% found in a meta-analysis [13]. The biggest difference between efficacy and AEs (Fig. 2) was observed at the 2.5 mg dose which is therefore the recommended dose for zolmitriptan [15].

Oral naratriptan apparently has a dose–response curve for efficacy [16] with a plateau which starts at 7.5 mg (Fig. 4). For AEs there is a similar plateau in this dose range. At 2.5 mg there are no more AEs than with placebo, as has also been observed in a meta-analysis [13]. The 2.5 mg dose of naratriptan was subsequently chosen as a recommended dose without any more AEs than placebo, the so-called “gentle triptan” [17].

**Fig. 5:**
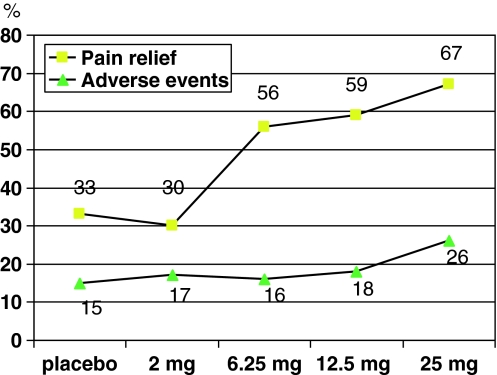
Effect of almotriptan 2, 6.25, 12.5, and 25 mg on headache relief and adverse events in one RCT [18]

The dose–response curves for almotriptan are shown in Fig. 5 [18] and there is a slight increase in efficacy from 6.25 (56%) to 25 mg (66%). The incidences of AEs are remarkably low and first at 25 mg there is a slight increase compared with placebo. The AEs up to 12.5 mg (16–18%) were described as being mild in the majority of patients whereas the AEs after 25 mg (25%) were described as being of moderate intensity in 48% of cases. Also in a meta-analysis almotriptan 12.5 mg was found to have AEs at the placebo level [13]. Mostly based on the change in intensity of AEs almotriptan 12.5 mg was chosen as the recommended dose [15, 18].

**Fig. 6:**
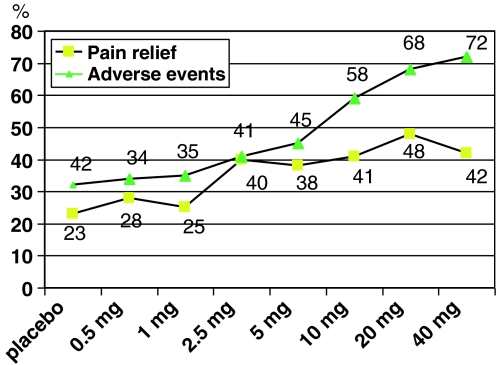
Effect of frovatriptan 0.5, 1, 2.5, 5, 10, 20, and 40 mg on headache relief and adverse events in two RCTs [19]

The efficacy of frovatriptan was evaluated by pooling the results of two RCTs [19]. The combined results are shown in Fig. 6. From 2.5 mg and with higher doses there is a flat dose–response curve. Below 2.5 mg there is no efficacy. The incidences of AEs increase with dose and there is a maximum of 72% at 40 mg. The recommended dose is frovatriptan 2.5 mg, the lowest dose with efficacy.

For rizatriptan and eletriptan the total incidences of AEs (any patients with an AE) are not reported but the incidences of individual AEs are given in tables [8–11]. Thus only the dose–response curves for efficacy of these two triptans can be evaluated. In one dose-finding RCT (*n* = 417) headache relief was 18% with placebo, and 21, 45, and 48%, with rizatriptan doses of 2.5, 5, and 10 mg, respectively [10]. In a RCT (*n* = 449) exploring the upper part of the dose–response curve for rizatriptan headache relief was 18% with placebo and 52, 56, and 67% with 10, 20, and 40 mg doses of rizatriptan. AEs occurred more frequently after a 40 mg dose of rizatriptan [11]. In one RCT (*n* = 1,190) investigating the effect of eletriptan headache relief was 20% with placebo an 47, 62, and 59% with 20, 40, and 80 mg doses of eletriptan [9] and in another RCT (*n* = 1334) [8] headache relief was 22% with placebo and 47, 62, and 59% with the eletriptan doses of 20, 40, and 80 mg, respectively. In both RCTs AEs were comparable for eletriptan 20 mg and placebo [8, 9]. AEs from different trial programmes are difficult to compare because of differences in the methodology of collecting AEs. In a meta-analyses any AE (placebo-subtracted) were 7 and 13% after 5 and 10 mg doses of rizatriptan; and 2, 6, and 18% after 20, 40, and 80 mg, respectively, doses of eletriptan [13]. There is thus also for these two triptans an increase in the incidence of AEs with increase in doses.

**Fig. 7:**
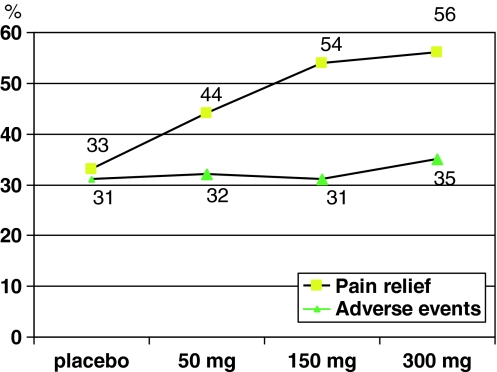
Effect of telcagepant 50, 150, and 300 mg on headache relief and adverse events in one RCT [21]

Telcagepant, a calcitonin gene-related peptide (CGRP) receptor antagonist, is currently being developed for the acute treatment of migraine. In one small dose-defining RCT [20] doses of 300 and 600 mg telcagepant were found comparable and the 300 mg dose was selected for further investigation. The dose–response curves for telcagepant in doses from 50 to 300 mg are shown in Fig. 7 [21]. The incidence of AEs is at the placebo level, confirming the lack of CGRP antagonists on human vasculature [22], and there is probably a plateau for efficacy from 150 or 300 mg and further up [21, 23]. The recommended dose will probably be 300 mg telcagepant, a dose with maximum effect and AEs on placebo level.

## Discussion

In 2002, it was stated that triptans have served as the foot soldiers or the advances in migraine research during the latter part of the twentieth century [24]. How effective are these revolutionary drugs then in clinical practice?

The triptans are per se highly effective drugs confer the 85–91% headache relief at 2 h after subcutaneous sumatriptan and naratriptan [1–3]. Theoretically, it should be possible by increasing the oral dose of a triptan to obtain similar high response rates. This is, however, not the case. Even with similar plasma concentrations of the sumatriptan and naratriptan after oral and subcutaneous administration the injection is still superior to the oral form [4]. As shown in Figs. 1, 2, 3, and 5 there is for several triptans, sumatriptan, zolmitriptan, and frovatriptan, a flat upper part of the dose–response curves. In addition, the efficacy even with very high doses, e.g., the 40 mg dose of frovatriptan. (42%) and of rizatriptan (67%), is not near the efficacy of the subcutaneous form, vide supra. This higher efficacy of injected triptans compared with the oral form is most likely due to a quicker rise in blood concentrations after subcutaneous injections [4].

The upper part of the dose–effect curves for several triptans, sumatriptan, zolmitriptan, and frovatriptan (Figs. 1, 2, 3, and 6) demonstrate a ceiling effect for response on migraine pain. This ceiling effect is especially pronounced for frovatriptan for which a 16-fold increase to 40 mg from the 2.5 mg dose did not result in an increase in efficacy (see Fig. 6). In contrast the dose–response curves for AEs show that the incidence of AEs increases with increasing doses (Figs. 1, 2, 3, 4, 5, and 6), and there is no indications of a ceiling effect.

Only reporting the incidence of AEs does not in all cases give the full picture of the clinical impact of the AEs. Thus for almotriptan 12.5 mg AEs were reported as mild whereas for 25 mg they were reported as moderate [18]. The global impact of AEs should be measured on suitable quality of life scales in the future [25].

Compared to the traditionally used drug, ergotamine, which in addition to its 5-HT1B/1D has agonistic effect on e.g., the dopamine D_2_ receptor [26], the triptans act selectively on the 5-HT1B/1D receptor [15, 27] and should thus have a better tolerability profile than ergotamine. Thus in one RCT rectal ergotamine 2 mg (73%) was slightly superior to rectal sumatriptan 25 mg (63%) for headache relief but caused significantly more nausea and/or vomiting: 28 and 7%, respectively [15, 28].

Even if just recording the incidence of AEs in the balance between efficacy and tolerability is not the ideal measure of tolerability it is fair measure for the potential for AEs of a triptan in the migraine population and in several cases the incidence of AEs has determined the recommended doses of the triptans. The recommended doses are in most cases a realistic compromise between efficacy and tolerability.

The new CGRP antagonist telcagepant has an excellent tolerability with AEs on the placebo level (see Fig. 6 [21, 23]). Telcagepant has a headache relief of 56% and has a 26% pain-free response [29] which is lower than 40% for rizatriptan 10 mg [13].

## Clinical comments

My personal rating of the triptans and telcagepant is given in Table 1. It is based both on comparative RCTs [5], two systematic reviews [27, 30], and a meta-analysis [13]. For efficacy + is given for a drug somewhat better than placebo, ++ is given for an effective drug, and +++ for a highly effective drug. For tolerability 0 is given for no more AES than placebo, + for <10% more AEs than placebo, ++ for <25% more AEs than placebo, and +++ for >25% more AEs than placebo.

**Table 1:** Efficacy and tolerability of triptans and telcagepant

Drug and dose (mg)	Efficacy (+, ++, and +++)	Adverse events potential (0, +, ++, and +++)
Subcutaneous sumatriptan: 6	+++	+++
Sumatriptan: 50	++	+
Sumatriptan: 100	++	++
Naratriptan: 2.5	+	0
Rizatriptan: 10	++	++
Zolmitriptan: 2.5	++	++
Eletriptan: 40	++	++
Almotriptan: 12.5	++	0
Frovatriptan: 2.5	+	+
Telcagepant: 300	++	0

For explanation of (+ to +++) for efficacy and of (0 to +++) for AES potential, see text. The rating is based on [13, 15, 21, 23, 27, 30]

It should be noted that there are most likely inter-individual difference to responses to triptans. Thus one patient A may use one triptan successfully whereas patient B may prefer another triptan. This variability among triptans is most likely due to both a pharmacokinetic and pharmacodynamic variability among the drugs [31]. From a pharmacokinetic point of view almotriptan has the advantage of a high oral bioavailability of 80% and is more unlikely to vary among subjects than e.g., sumatriptan with an oral bioavailability of 14% [15, 27]. Because of no more AEs in RCTs than placebo (see Fig. 5) almotriptan 12.5 mg can apparently (see Table 1) be a first choice triptan if no AEs are tolerated. It should be noted, however, that some patients can experience so-called “triptan” symptoms (see below) even after almotriptan as after other triptans. Sumatriptan is now of patent in most countries and sumatriptan 50–100 mg should therefore in clinical practice be the triptan of first choice when triptans are used de novo in migraine patients.

Even if the AEs after triptans are in most cases mild to moderate and transient they can be frightening for the patients which should be informed about possible AEs. Somnolence and asthenia are reported as AEs of triptan but they are most likely partly treatment-emergent CNS symptoms of the migraine attack following the treatment with triptans [26]. Even so they are experienced by the patients as bothersome AEs. The so-called “triptans” symptoms [32] are shown for placebo and 2.5 mg recommended dose of zolmiriptan in Table 2 [15, 33]. Note that zolmitriptan 2.5 mg caused 17% more adverse events than placebo. Chest symptoms (mainly tightness and pressure) have been reported to occur in up to 20% (tablets) and 40% (subcutaneous injection) of the patients treated with sumatriptan some time [15, 34]. Such symptoms can be a frightening experience for the patients, and they should be warned in advance of the risk of the symptoms and should be informed about the transient and generally benign nature.

**Table 2:** Incidence, most common AEs, chest-related AEs in placebo-controlled RCTs after oral administration of zolmitriptan 2.5 mg [15, 33]

	Placebo (*n* = 401) (%)	Zolmitriptan 2.5 mg (*n* = 498) (%)
Patients with at least one AE	117 (29)	227 (46)
*Most common AEs*		
Asthenia	13 (3)	16 (3)
Dry mouth	7 (2)	16 (3)
Nausea	15 (4)	45 (9)
Dizziness	16 (4)	42 (8)
Somnolence	12 (3)	30 (6)
Paresthesia	6 (1)	21 (4)
Warm sensitation	7 (2)	21 (4)
*Chest-related AEs*		
Chest tightness	2 (<1)	13 (3)
Chest pain	1 (<1)	1 (1 < 1)
Chest heaviness	0 (0)	1 (<1)
Chest pressure	1 (<1)	1 (<1)

If telcagepant becomes available it will be the drug of first choice for the patients with migraine and cardiovascular diseases or high risk for such diseases. It will also be a good choice if the migraine patient has intolerable AEs when treating with triptans.

It should be noted that with any drug used in acute migraine treatment there is a different balance of efficacy and tolerability in the individual patient and there is thus no standard dose that suites every patient. In addition, some patients may prefer a very effective drug with some AEs to a drug with lower efficacy and virtually no AEs. Drugs and doses should thus be tailored to the need of the individual patient.

Finally, it is important to note that the majority of the patients experience no AEs with use of the oral specific 5HT1B/1D receptor agonists, the triptans, in the recommended doses (see, Fig 1, 2, 3, 4, and 5).

When AEs occur they are in most cases mild to moderate and transient. On balance, the triptans with their proven efficacy and an acceptable tolerability profile have been a major step forward in the acute treatment of migraine.
